# Amide Bond Activation of Biological Molecules

**DOI:** 10.3390/molecules23102615

**Published:** 2018-10-12

**Authors:** Sriram Mahesh, Kuei-Chien Tang, Monika Raj

**Affiliations:** Department of Chemistry and Biochemistry, Auburn University, Auburn, AL 36849, USA; mzs0154@auburn.edu (S.M.); kzt0026@tigermail.auburn.edu (K.-C.T.)

**Keywords:** peptide bond cleavage, amide bond resonance, twisted amides, enzymes, metal complexes, catalysts

## Abstract

Amide bonds are the most prevalent structures found in organic molecules and various biomolecules such as peptides, proteins, DNA, and RNA. The unique feature of amide bonds is their ability to form resonating structures, thus, they are highly stable and adopt particular three-dimensional structures, which, in turn, are responsible for their functions. The main focus of this review article is to report the methodologies for the activation of the unactivated amide bonds present in biomolecules, which includes the enzymatic approach, metal complexes, and non-metal based methods. This article also discusses some of the applications of amide bond activation approaches in the sequencing of proteins and the synthesis of peptide acids, esters, amides, and thioesters.

## 1. Introduction

**Figure 1:**
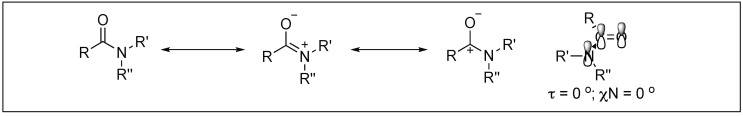
Classical amide bond resonance.

The amide bond is one of the most abundant chemical bonds and widely exists in many organic molecules and biomolecules [1,2,3,4,5,6]. Nature has used amide bonds to make these important biomolecules because of the high stability of amide bonds towards various reaction conditions (acidic and basic conditions), high temperature, and the presence of other chemicals [7]. The high stability of amide bonds is attributed to its tendency to form a resonating structure, which provides a double bond character to the amide CO-N bond (Figure 1) [8,9,10]. The resonance of these amide bonds forms a planar structure and hinders the free rotation around the CO-N bond, thus, it is responsible for 3D structures adopted by proteins and other biomolecules. These 3D structures of biomolecules are responsible for various important biological functions.

Hansen et al. carried out the rate studies on the hydrolysis of amide bonds at various pH conditions [11]. The study concluded that at pH 7, the rate of hydrolysis is due to the direct attack of water on peptide and measured as kH_2_O. The rate constant showed that the half-life of the amide bonds is 267 years, similar to the value determined by Radzicka and Wolfenden [12]. This study also showed that the rates of acid (kH_3_O^+^) and base hydrolysis (kOH^−^) are identical, therefore, the rate of the hydrolysis of the peptide bond is dominated by kH_2_O throughout the pH range from pH 5–9. 

**Figure 2:**
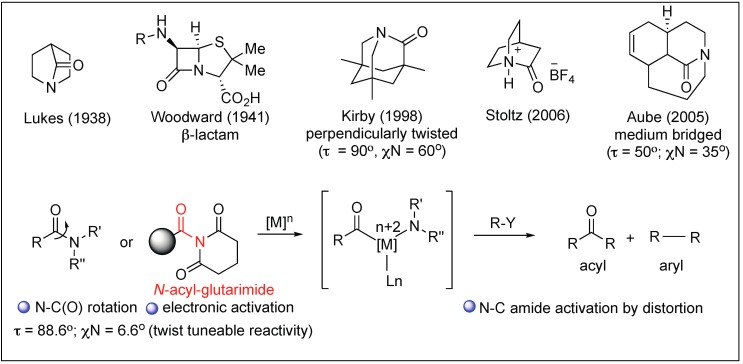
Twisted amides for activation of amide bonds.

Recently, various methods have been reported in the literature to activate the amide bonds towards a variety of nucleophiles or electrophiles for the synthesis of other organic compounds. This includes the use of enzymes, metal complexes, and non-metal based methods [13,14,15]. One widely reported approach for the activation of amide bonds involves the distortion of amide bonds, thus, the amide bond is no longer able to form a resonating structure, loses its double bond character, and becomes more susceptible to nucleophilic or electrophilic attack. A higher distortion of the amide bond from the planar structure makes it more reactive, as evidenced by various twisted amide bonds present in cyclic nonplanar bridged lactams, as demonstrated by Stoltz [16,17], Kirby [18,19,20], and others [21,22,23] (Figure 2). One of the special cases to achieve maximum rotational inversion of the amide bond so that it remains in the twisted conformation is the use of *N*-acyl-glutarimides [24,25,26,27,28,29] and *N*,*N*-substituted amide bonds [30,31] (Figure 2). It is this strong distortion of amide bonds that provides amide bonds with a high reactivity toward a variety of nucleophiles and electrophiles. 

There are already some excellent review articles in the literature covering the reactivity of twisted/activated amide bonds for the synthesis of the variety of different organic molecules such as ketones, esters, acids, and alcohols, by cross-coupling reactions [24,25,26,27,28,29,30,31]. The main focus of this review is to summarize the methods for the activation of less reactive amide bonds present in biomolecules such as peptides, proteins, glycopeptides, nucleotides in DNA and RNA and various other peptide bioconjugates, toward attack by various nucleophiles. This task was accomplished by various methods such as by using biological molecules, metal complexes, and non-metal based methods and is discussed below. 

## 2. Biomolecules for the Activation of Amide Bonds—The Enzyme-Directed Hydrolysis of Amides

We have summarized different kinds of enzymes, their mechanisms of hydrolysis of unactivated peptide bonds, and the point of cleavages in Table 1.

### 2.1. Serine Proteases

Amide bonds are widely present in proteins due to their high stability and the tendency of amide bonds to exist in resonating structures, which is one of the key factors responsible for secondary structures adopted by proteins and their biological activities. Nature has developed some methods for the cleavage of highly stable amide bonds to control their functions. One such approach is the use of enzymes (serine proteases), which have active sites, and binding pockets for binding to particular amino acids followed by the activation of amide bonds for hydrolysis. These enzymes exist in various families such as trypsin, chymotrypsin, elastase, subtilisin, etc., but have a similar catalytic site containing oxyanion binding hole with Ser, His and Asp triad [32,33]. Some of the proteases have catalytic dyads with two amino acids at the active site, however, triads are the most common.

**Figure 3:**
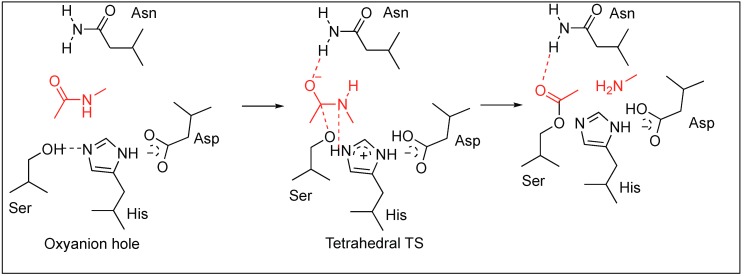
General pathway of serine proteases directed amide bond hydrolysis.

All these enzymes based on the binding pocket prefer to bind to particular amino acids but the mechanism by which they hydrolyze the amide bond is similar. During the catalysis, these enzymes form an oxyanion hole made up of three amino acids—His, Asp, and Ser—which work in a synergistic manner to break the amide bond (Figure 3). First, the side chain of Asp makes a hydrogen bond with histidine, thus making it more nucleophilic. Second, histidine forms a strong H-bond with the hydroxyl group of serine and abstracts the proton from the hydroxyl group (OH) of serine which in turn attacks amide bond to form a tetrahedral transition state (TS). This TS eventually collapses resulting in the hydrolysis of the amide bond by acid–base catalysis. Wells et al. demonstrated the importance of these residues at the active site by mutating it to alanine [32,33]. They showed that any mutation in the catalytic triad greatly reduces the turnover number which is a consequence of the changes in the enzyme mechanism. Residues in the catalytic triad function in a strongly synergistic manner and contribute a factor of 2 × 10^6^ to the rate enhancement. The study concluded that enzymes increase the rate of amide bond hydrolysis at by least 10^9^ to 10^10^ times that of the non-enzymatic hydrolysis of amide bonds.

### 2.2. Cysteine Proteases

Cysteine proteases (CPs) hydrolyze the peptide bonds with maximum efficiency at pH 4–6.5 [14]. The thiol group of cysteine protease is susceptible to oxidation so the environment of the enzyme is reducing in nature. Till now 21 families of CPs have been discovered [34,35,36,37]. CPs form a triad at the active site during the hydrolysis of the peptide bond made up of Cys-His-Asn residues. First, Asn forms the hydrogen bond with His, then His abstracts the proton from Cys to generate a nucleophilic thiolate ion (S^−^) similar to enolate ion generated by serine proteases (Figure 3). Next, the thiolate ion (S^−^) attacks the carbonyl group of the peptide resulting in the formation of a tetrahedral intermediate TS followed by the hydrolysis of the amide bond [34,35,36,37]. 

### 2.3. Metalloproteases

Metalloproteases are members of a class of proteases that require a metal ion cofactor at the active site for the hydrolysis of peptide bonds [38]. The most common metal ion cofactor present in metalloproteases is the zinc ion (Zn^2+^) [39]. Other transition metals such as Co^2+^ and Mn^2+^ are capable of restoring the functions in zinc-metalloproteases where the Zn^2+^ core has been removed [39]. Metalloproteases are divided into two major families: metalloendopeptidases and metalloexopeptidases. The names of these families are based on the site of the hydrolysis of the peptide bonds [40,41]. Metalloendopeptidases cleave the internal amide bonds whereas metalloexopeptidases cleave the amide bonds present at the C- or N-terminus of peptides.

#### 2.3.1. Metalloendopeptidase: Thermolysin

Thermolysin (TLN) catalyzes the cleavage of the internal peptide bond at the amino-side of large hydrophobic amino acids, such as leucine, isoleucine, or phenylalanine. TLN and TLN-like proteins require Zn^2+^ as a metal ion cofactor for the cleavage of amide bonds [42,43,44,45,46,47].

**Figure 4:**
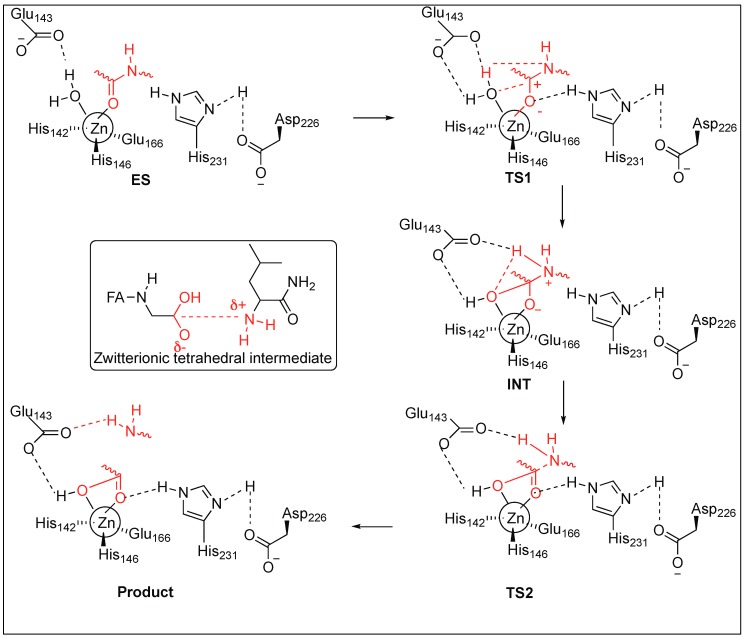
Thermolysin Mechanistic pathway.

TLN-mediated hydrolysis of the peptide bond is a two-step process (Figure 4) [48,49,50,51]. The active site of TLN contains three residues—His142, His146, Glu166—and a water molecule, which are bound to the Zn^2+^ ion. First, the carbonyl group of the peptide coordinates with Zn^2+^ and displaces the hydrogen of a water molecule to form an H-bond with Glu143 and the oxygen of the water molecule remains associated to the Zn^2+^ ion, resulting in the formation of the enzyme-substrate complex (ES). Second, the oxygen of the water attached to Zn^2+^ attacks the carbonyl carbon of the peptide, resulting in the formation of transition state 1 (TS1). TS1 is stabilized by the formation of the H-bond with Asp226 and His231 at the carbonyl oxygen of the peptide followed by the formation of intermediate gem-diolate (INT) by the breakage of hydroxyl OH bond of water. The amide of the peptide forms a hydrogen bond with the H of H_2_O. Third, the carbonyl bond rearrangement in TS2 leads to the breakage of the amide bond (CONH) of the peptide and releases the N-terminal peptide. The rate-determining studies showed that the collapse of a zwitterionic tetrahedral intermediate (INT) is a rate-limiting step (Figure 4).

#### 2.3.2. Metalloexopeptidase: Carboxypeptidase A

**Figure 5:**
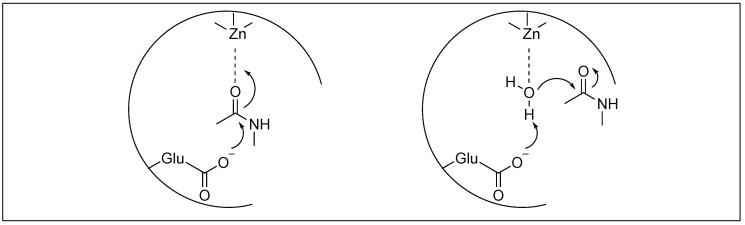
Mechanisms of carboxypeptidase A.

Carboxypeptidase A (CPA) is a 35 kDa metalloenzyme and contains a Zn^2+^ ion cofactor in its active site [52,53,54]. CPA is an exopeptidase, which catalyzes the hydrolysis of amide bonds present at the C-terminus comprising large hydrophobic side chains. Two different mechanisms have been proposed for the cleavage by these metalloproteases (Figure 5) which showed the importance of Lewis acid catalysis for the activation of amide bonds [53,54]. One involves the Lewis-acid activation of the carbonyl group of the amide bond by Zn^2+^, followed by the attack of water (Figure 5). The second involves the Lewis-acid activation of H_2_O by Zn^2+^ ions followed by the attack of the hydroxide ion of the water on the carbonyl group of the amide bond (Figure 5). 

#### 2.3.3. Glutamate Glycosylation for Amide Bond Cleavage

**Figure 6:**
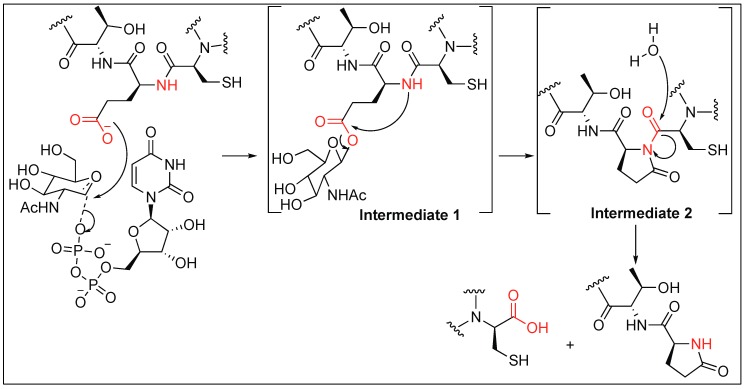
Glycosylation pathway.

Human enzyme *O*-GlcNAc transferase (OGT) is essential for the cleavage of amide bonds in host cell factor-1 (HCF-1). HCF-1 cleavage takes place at the N-terminal glutamic acid by the glycosylation which is catalyzed by enzyme OGT. Mechanistic studies showed that the glycosylation of the glutamate side chain (intermediate 1, Figure 6) leads to the formation of an enzyme-catalyzed internal pyroglutamate formation (intermediate 2, Figure 6) with the amidic nitrogen of the peptide backbone chain, which then undergoes spontaneous hydrolysis (Figure 6) [55]. Detailed mechanistic studies showed that the rate of conversion of glycopeptide to internal pyroglutamate was an order of magnitude slower than observed in the presence of OGT, thus, it was concluded that both the first and second steps occurred while the peptide is bound to OGT (Figure 6). Hydrolysis likely occurs after dissociation from the enzyme. It has also been reported that glycosylation on Thr next to glutamate also prevents the cleavage at the glutamate (Glu) because of the steric hindrance and thus the enzyme is unable to carry out the glycosylation of glutamate [56].

#### 2.3.4. Nicotinamidase (Pnc1) for the Hydrolysis of the Amide Bond of Nicotinamide

**Figure 7:**
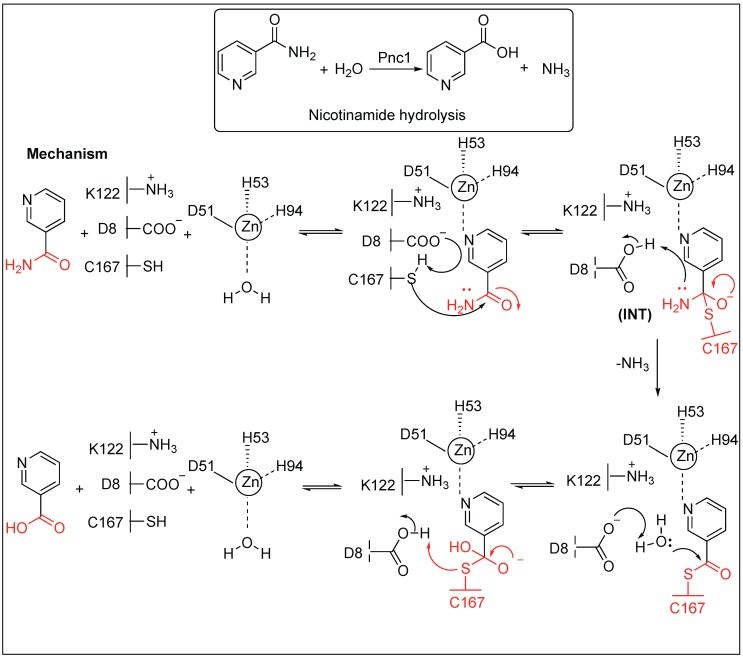
Mechanistic pathway of Pnc1 for hydrolysis of nicotinamide.

Nicotinamidases catalyze the cleavage of nicotinamide, which is a critically important part of NAD^+^ and NADH, to nicotinic acid and ammonia (Figure 7). A detailed study showed that both the carbonyl oxygen and the ring nitrogen of nicotinamide are critical for binding to the nicotinamidases and reactivity [57,58,59,60]. 

Three residues—Asp51, His53, and His94—in nicotinamidase (Pnc1) directly coordinate with Zn^2+^ at the active site and three other residues act as a catalytic triad (Cys167, Asp8, and Lys122) (Figure 7). In the first step, the substrate binds to the Zn^2+^ by nitrogen of pyridine ring and displaces the water molecules ligated to the Zn^2+^. Next, Asp8 removes the proton from Cys167, forming a thiolate, which, in turn, react with the amide carbonyl carbon of nicotinamide, leading to the formation of a tetrahedral intermediate (INT). The tetrahedral intermediate collapsed, resulting in the breakage of the amide bond and release of the ammonia. This is followed by the release of the nicotinic acid from the active site of the enzyme by acid-base catalysis.

#### 2.3.5. Flavoenzyme-Mediated Hydrolysis of the Amide Bond

**Figure 8:**
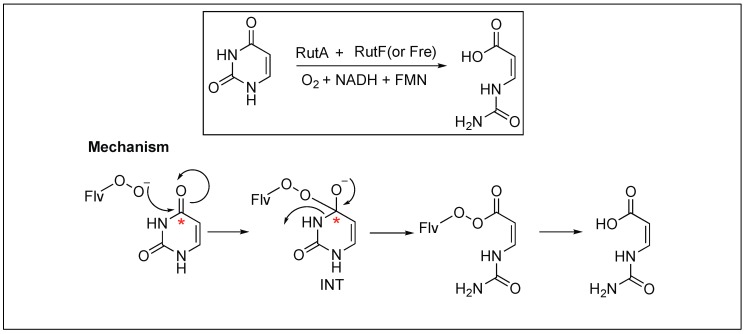
Flavoenzyme-mediated hydrolysis of amide bond.

Begley et al. demonstrated the role of flavoenzyme in the cleavage of the unactivated amide bond in uracil, a building block for RNA (Figure 8) [61,62,63]. The detailed mechanistic analysis showed that the reaction takes place through the oxidative mechanism that is initiated by the addition of a flavin hydroperoxide to the C* carbonyl of uracil, forming a tetrahedral intermediate (INT) (Figure 8). This is followed by the collapsing of the tetrahedral intermediate (INT), leading to the cleavage of an amide bond in uracil. This was the first example where such chemistry was shown by flavin hydroperoxides.

#### 2.3.6. Primary Amide Bond Hydrolysis by Antibodies

**Figure 9:**
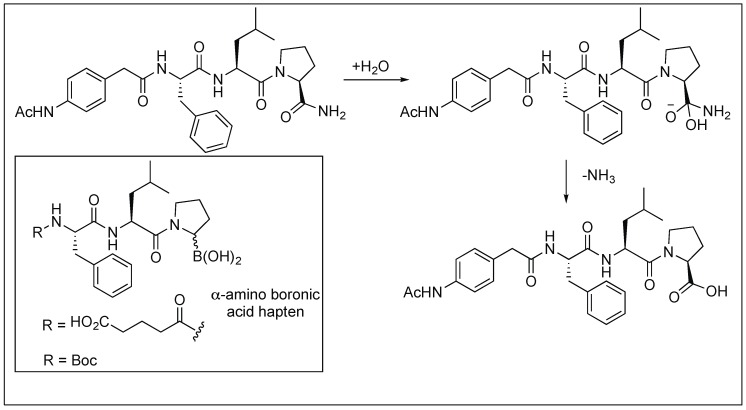
Antibody Fab catalyzed primary amide bond hydrolysis.

The antibody Fab-BL125 catalyzes the hydrolysis of the unactivated primary amide bond of the l-isomer of peptides to generate free peptide acid (Figure 9). The antibody showed high regio- and diastereoselectivity since the d-proline primary amide diastereoisomer did not undergo any hydrolysis. The antibody Fab-BL125 decreases the half-life of the peptide from 17.5 years to only 3.9 h. Such an antibody was obtained by using the α-amino boronic acid Hapten by a direct selection strategy from the antibody combinatorial libraries [64,65].

#### 2.3.7. RNA-Assisted Cleavage of Amide Bonds

**Figure 10:**
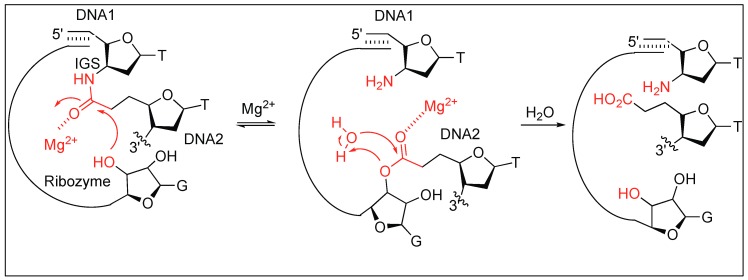
RNA catalyzed amide bond cleavage.

A group I RNA obtained by in vitro evolution catalyzes the cleavage of unactivated alkyl amides of DNA analog. This includes substrates with an amide bond that joins either two DNAs, or a DNA with a short peptide. The RNA increases the rate of hydrolysis by more than 10^3^ in comparison to the uncatalyzed reaction. The RNA-catalyzed amide bond cleavage was entirely dependent on Mg^2+^ where Mg^2+^ acts as a Lewis acid thus activating the carbonyl group of the amide bond for the nucleophilic attack by the hydroxy group of RNA (Figure 10). No amide bond cleavage was detected in the presence of other metal ions such as Zn^2+^, Ca^2+^, or Sr^2+^. A trace amount of amide bond cleavage was observed in the presence of MnCl_2_ which is in contrast to the RNA and DNA cleavage reactions [66].

To determine the generality of the amide bond cleavage by RNA, a series of substrates were explored. If a short DNA is attached to a peptide or an amino acid by using an amide bond, the immediate cleavage at the amide bond was observed in the presence of the RNA. The cleavage of the amide bond between the amino acid residues was not observed.

## 3. Metal Complexes for the Activation of Amide Bonds 

We have summarized different kinds of metal-based complexes, their mechanisms of hydrolysis of unactivated peptide bonds and point of cleavages in Table 2.

**Figure 11:**
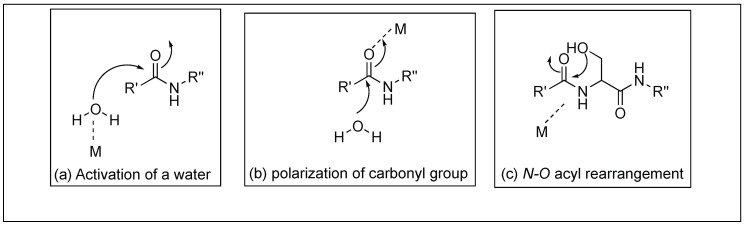
Metal-assisted peptide bond hydrolysis.

Another approach for the cleavage of peptide bonds involves the use of metal complexes. This metal complex has potential applications in the field of chemical biology, biochemistry, and bioengineering. A variety of metal ion complexes has been utilized for testing the reactivity with substrates such as peptides, and proteins [67]. Most of the metal catalyzed reactions reported so far are based on the activation of amide carbonyl or water by the Lewis acid mechanism of the metal ion. Another metal ion hydrolysis mechanism involves the formation of a square planar complex of the metal ions Cu(II), Ni(II), or Pd(II) with the Ser/Thr-His or Ser/Thr-Xaa-His sequence leading to the N→O rearrangement of the acyl moiety resulting in the cleavage of the peptide bond (Figure 11). In this section, we will provide few examples of both Lewis acid and the N→O acyl rearrangement for the cleavage of peptide bonds.

### 3.1. Lewis Acid Mediated Hydrolysis

#### 3.1.1. Simple Metal Ions

Yashiro et al. reported that the rate of hydrolysis of peptide bonds increases by almost all the metal ions and the highest conversion was observed for Zn(II) [68,69,70,71,72]. The active intermediate was metal complexes where metal binds to both the carbonyl group and the N-terminal amino group of the peptide bond. Cleavage of the peptide bond in the presence of the metal takes place through the mechanism in Figure 11b.

#### 3.1.2. Oxo Metal Ions

Parac-Vogt et al. proved that polyoxometalates oxo-metal compounds such as MoO_2_^−4^, WO_2_^−4^, CrO_2_^−4^, and VO_2_^−4^ cleave the peptide bonds in various dipeptides [73,74,75,76,77]. They have shown that these oxo-metal compounds also hydrolyze the amide bonds in their regular oligomeric forms, polyoxometalates (POMs). A POM is a unit where one or more atoms can be replaced by a metal center leading to the change in the coordination properties of POM. POMs were utilized for the Zr(IV)- and Ce(IV)-assisted peptide bond hydrolysis because they are homogenous in nature [78,79].

##### Zirconium Complex Mediated Hydrolysis of Peptide Bonds

**Figure 12:**
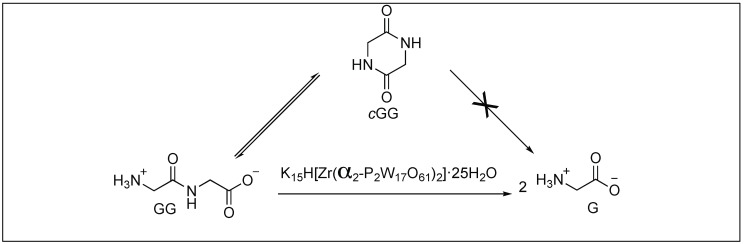
Peptide hydrolysis catalyzed by a polyoxometalate complex.

Parac-Vogt et al. reported the hydrolysis of peptide bonds catalyzed by a polyoxometalate complex for the first time. They demonstrated the role of metal-substituted Wells-Dawson type polyoxometalates K_15_H[Zr(α_2_-P_2_W_17_O_61_)_2_]·25H_2_O for the hydrolysis of peptide bonds in diglycine, triglycine, tetraglycine, and pentaglycine (GG), yielding glycine as a final product (Figure 12) [80]. A detailed mechanistic investigation by NMR showed that the free amino terminus and both carbonyl functionalities of GG interact with polyoxometalates K_15_H[Zr(α_2_-P_2_W_17_O_61_)_2_]·25H_2_O either by the formation of metal ion coordination complex or by the non-covalent interactions of the protonated amino group with the negatively charged surface of POMs, thus responsible for the activation of amide bonds towards hydrolysis. These POMs selectively cleave the C-terminal amide bond of glycylglycyl amide (GGNH_2_), resulting in the formation of GG. No free glycine amide (GNH_2_) was detected during the course of the reaction.

Recently, Vogt et al. utilized the MOF-808, a Zr(IV)-based metal−organic complex for the hydrolysis of the peptide bond in a wide range of peptides and proteins such as hen egg white lysozyme (HEWL) under physiological conditions [81,82]. The MOF-88 is heterogeneous in nature and is thus a reusable catalyst. The experimental studies and calculations showed that MOF-808 hydrolyzed the Gly-Gly bond by the formation of the active complexes with two adjacent Zr(IV) centers of the {Zr_6_O_8_} core by coordination with amide oxygen and the amine nitrogen atoms. The catalytic efficiency of MOF-808 towards the hydrolysis of peptides is dependent on the bulkiness and nature of the side chain amino acid residues. Dipeptides with small or hydrophilic residues undergo cleavage faster as compared to those with bulky and hydrophobic residues. 

##### Asp-Xaa Selective Hydrolysis of the Peptide Bond by Oxo-Metal Ions

**Figure 13:**
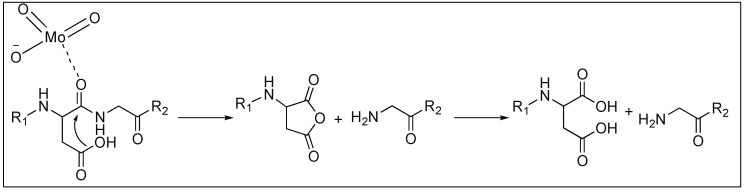
Oxomolybdate(VI) catalyzed cleavage of peptide bonds.

It has been reported that oxomolybdate(VI) catalyzes the cleavage of various peptides containing aspartic acid (Asp) with cleavage at the C-terminal side of the Asp residue. This is due to the attack of the side chain of the Asp on the amidic carbonyl which is activated by the coordination with oxomolybdate(VI), resulting in the formation of the five-membered ring and simultaneous cleavage of the amide bonds (Figure 13) [81,82].

##### Various Other Metal Complexes

**Figure 14:**
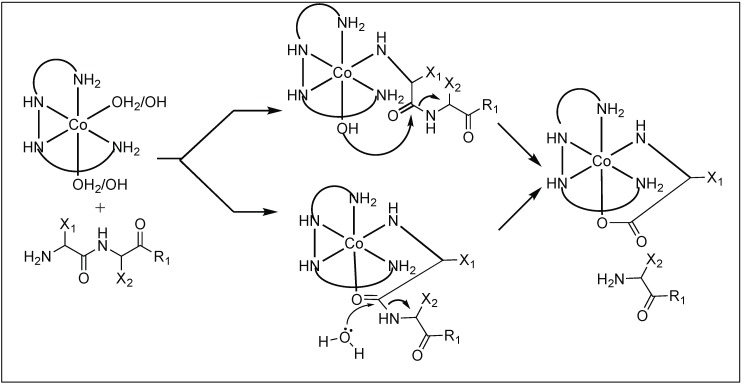
Co(III) Complex catalyzed peptide hydrolysis.

The Westheimer and Trapmann groups showed that the metal complexes containing Co(II), Cu(II), and Ni(II) ions cleave various dipeptides [83,84]. Out of various metal complexes, the Co(III) complex [Co(trien)OH(H_2_O)]^2+^ is one of the widely studied metal ion complexes and carried out the rapid hydrolysis of the peptide bond [85,86,87,88]. The detailed mechanistic investigation showed that first, the metal complex [Co(trien)OH(H_2_O)]^2+^ forms a tertiary complex with a dipeptide by the replacement of an equatorially coordinated water molecule in the octahedral Co(III) complex by the N-terminal amine of the peptide. This mode of coordination brings the axially bound hydroxyl group in close proximity to the peptide carbonyl resulting in the hydrolysis of the peptide bond (Figure 14).

##### Anchoring at Cys Side Chains: Molybdocene

**Figure 15:**
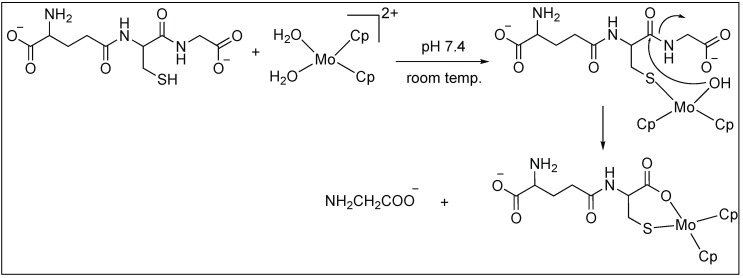
Mo catalyzed peptide hydrolysis.

Erxleben et al. showed that molybdocene dichloride, Cp_2_MoCl_2_, cleaves the amide bond at the C-terminal side of cysteine to generate Cys-Gly from Gly-Cys-Gly [89]. The cleavage is highly selective for the C-side of Cys because it leads to the formation of the favorable six-membered ring. The mechanism of this reaction is illustrated in Figure 15 [89].

#### 3.1.3. Anchoring at Met, His, and Cys Side Chains: Palladium(II) Complexes for the Cleavage of Amide Bonds

**Figure 16:**
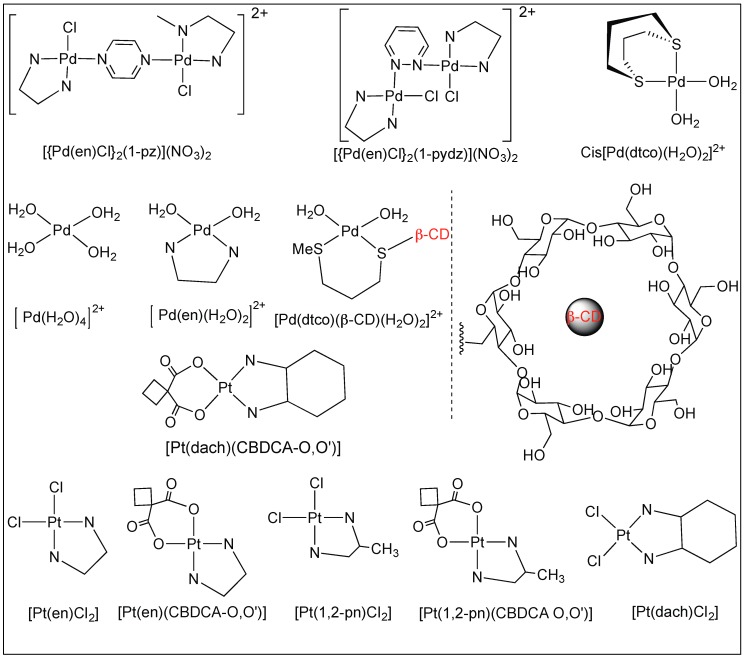
Pd and Pt complexes for activation of amide bonds.

Figure 16 shows the examples of platinum(II) and palladium(II) complexes which are known for the cleavage of amide bonds under mild conditions. These platinum(II) and palladium(II) complexes attach to the sulfur atom of cysteine, *S*-methylcysteine, and methionine in peptides, thus, promoting the selective cleavage of the unactivated amide bonds at the C-side of the amino acid (Figure 16) [90,91,92].

##### Pyrazine and Pyridazine Palladium(II)-Aqua Dimers

**Figure 17:**
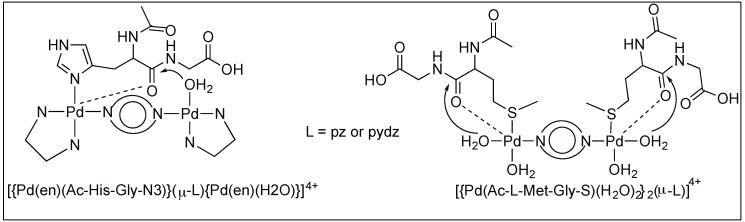
Pd(II) aqua dimers.

The complete hydrolysis of the amide bonds of peptides *N*-acetylated-l-histidylglycine (Ac-l-His-Gly) and -l-methionylglycine (Ac-l-Met-Gly) at the C-terminal side of Met and His in the pH range 2.0 < pH < 2.5 was catalyzed by two dinuclear palladium(II) complexes, [{Pd(en)Cl}_2_(l-pz)](NO_3_)_2_ and [{Pd(en)Cl}_2_(l-pydz)](NO_3_)_2_ at 37 °C. The hydrolysis is assisted by the formation of complexes between the side chains of methionine and histidine and the metal complexes (Figure 17) [93,94,95].

##### Platinum Complexes for the Cleavage of the Amide Bond

**Figure 18:**
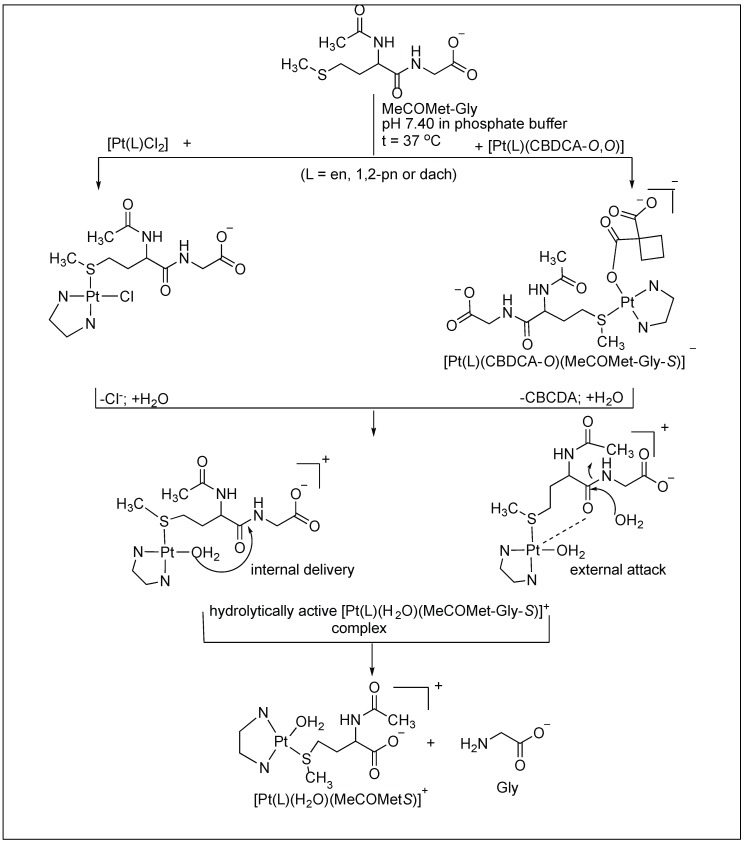
Hydrolytic reactions of Pt complexes.

^1^H-NMR investigation of the cleavage reactions between various Pt(II) complexes of the type [Pt(L)Cl_2_] and [Pt(L)(CBDCA-*O*,*O*′] (L = ethylenediamine-en; (±)-*trans*-1,2-diaminocyclohexane-dach; (±)-1,2-propylenediamine-1,2-pn and CBDCA is the 1,1-cyclobutanedicarboxylic anion) and the *N*-acetylated-l-methionylglycine dipeptide (MeCOMet-Gly) were reported by Djuran and co-workers [93,94,95,96]. The comparison of the rate studies of these Pt complexes for the cleavage of *N*-acetylated-l-methionylglycine dipeptide (MeCOMet-Gly) showed that the rate of hydrolysis decreases with the increase in the steric bulk of the CBDCA and chlorido Pt(II) complexes (en > 1,2-pn > dach) (Figure 18).

**Figure 19:**
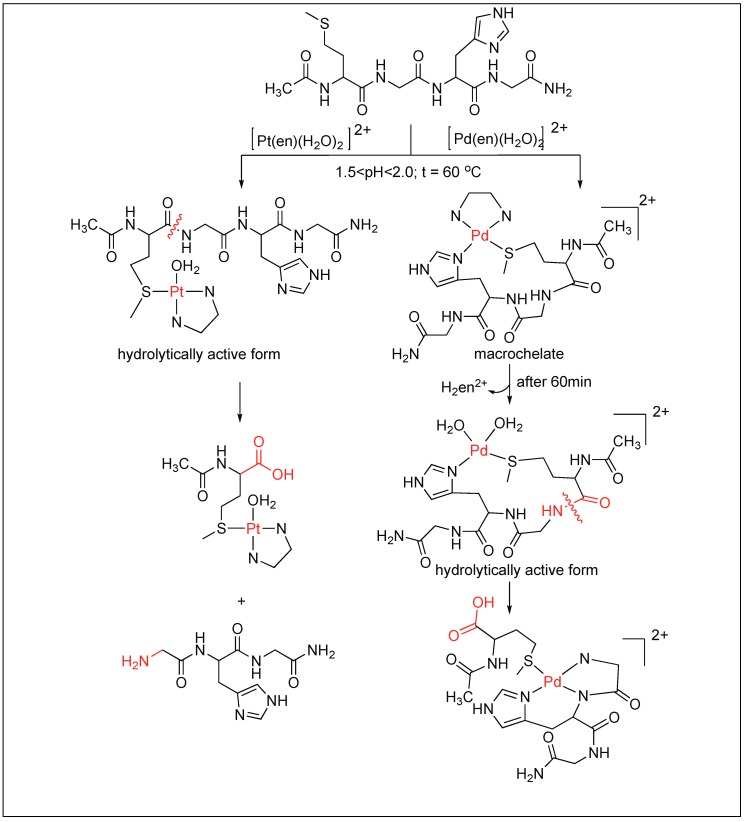
Hydrolytic reactions of Pd and Pt complexes.

Later, these [Pd(en)(H_2_O)_2_]^2+^ and [Pt(en)(H_2_O)_2_]^2+^ complexes were used for the cleavage of tetrapeptide (MeCOMet-Gly-His-GlyNH_2_) in the pH range of 1.5–2.0 and at 60 °C. The study showed that these complexes are highly selective for the cleavage of the amide bond at the C-terminal side of methionine (Figure 19) [97]. The high selectivity for the Met-Gly amide bond compared to the other amides is due to the high affinity of the Pt(II) and Pd(II) ions for the sulfur atom on Met. Two different mechanisms for the cleavage of tetrapeptide at the C-terminus of Met residue by Pd complexes have been proposed. One involves the direct coordination of the Pd complex with Met followed by the cleavage. The second involves the formation of macrochelate with both His and Met followed by the hydrolysis of the amide bond.

**Figure 20:**
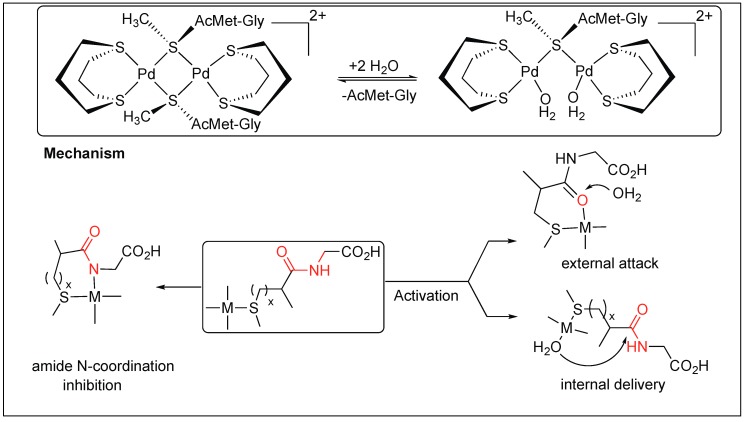
[Pd(dtco)(H_2_O)_2_]^2+^ mediated hydrolysis of amide bond.

Cis[Pd(dtco)(H_2_O)_2_]^2+^ leads to the selective cleavage of the amide bond at the C-terminal of Met (Figure 20) [98]. Coordination of the metal complex promotes hydrolysis by two different mechanisms. The first involves the formation of a six-membered complex by the chelation of a metal atom with both the sulfur of Met and the carbonyl of the amide bond, thus, activating the amide bond toward cleavage by an external attack from water. This mechanism is favorable in the case of platinum(II) promoters and substrates with smaller anchoring side chains. Second, the mechanism involves the chelation of metal with sulfur only, followed by the internal attack of water molecules to the amide bond. This mechanism is favorable with palladium(II) promoters and substrates with longer anchoring side chains (Figure 20) [98].

**Figure 21:**
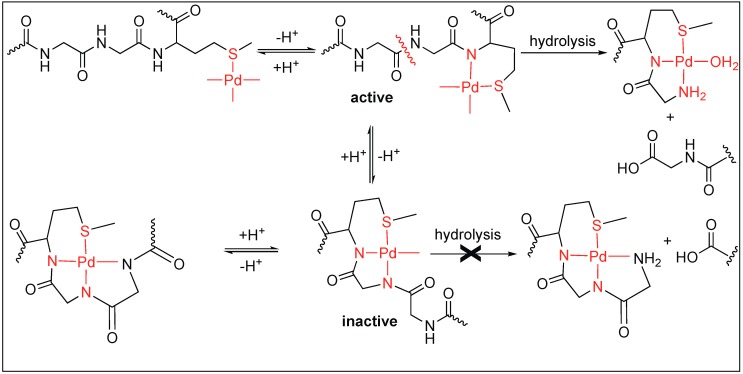
[Pd(H_2_O)_4_]^2+^ mediated hydrolysis of amide bond.

Kostic et al. utilized the Pd(H_2_O)_4_ complex for the cleavage of decapeptide (Ac-AKYGGMAARA) under acidic conditions (pH 2.3) at 60 °C [99]. The cleavage of the peptide bond took place at the Gly residue next to Met on the N-terminal side and generated two fragments after 24 h. The Pd(H_2_O)_4_ complex binds to the S of the Met residue leading to the formation of two different complexes under the reaction conditions (active and inactive), which are in equilibrium with each other (Figure 21). The active complex led to the formation of two fragments through hydrolysis but the inactive complex did not undergo any hydrolysis. The coordination of two amide nitrogen atoms in the inactive complex quench the Lewis acidity of Pd(II), thus no hydrolysis was observed.

**Figure 22:**
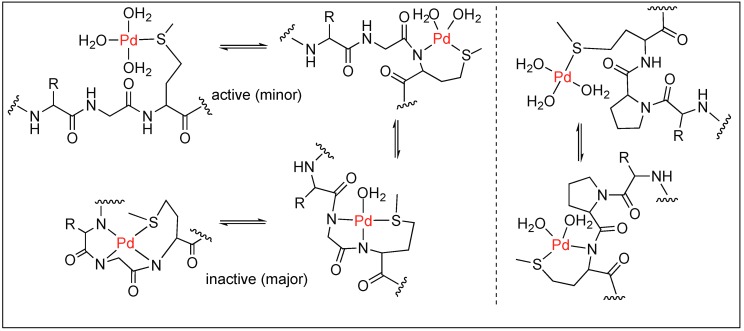
Pd triggered pH dependent hydrolysis of amide bond.

Next, Kostic et al. used this metal complex Pd(H_2_O)_4_ for the cleavage of the amide bond under neutral conditions in different peptide substrates such as Gly-Met, Sar-Met, and Pro-Met. This study showed that the rate of hydrolysis at a neutral pH is slow compared to that at a low pH [44]. Interestingly, the complete cleavage of Sar-Met and Pro-Met was observed but no cleavage was observed for Gly-Met at a neutral pH. This is due to the difference in the equilibrium position of the Gly-Met compared to that of Pro-Met. In Gly-Met, equilibrium is more shifted towards the inactive form at neutral pH due to the ability of Gly to form a strong coordinate bond with Pd. In contrast, for the peptide containing Sar-Met or Pro-Met, the equilibrium is shifted towards the hydrolytically active form because they are unable to form a strong coordination with Pd due to the tertiary amide of the Pro or Sar residue (Figure 22).

**Figure 23:**
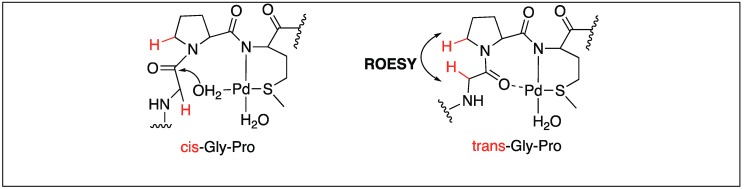
Cis/Trans conformations of Pd complexes.

In the case of Gly-Pro-Met, the hydrolysis of the amide bond can take place either through the external or internal attack of water molecules depending upon the cis/trans conformation adopted by proline (Figure 23). The ROESY NMR studies showed that the hydrolysis of the Gly-Pro-Met peptide takes place by the external attack of water on trans-Gly-Pro [100].

**Figure 24:**
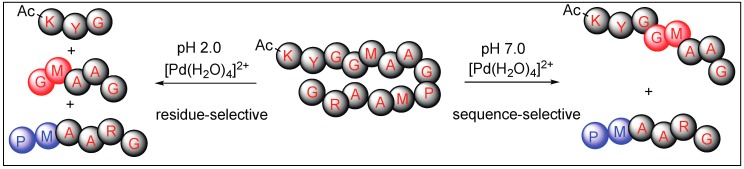
pH selective Pd catalyst for the hydrolysis.

Overall, the Pd(H_2_O)_4_ complex is residue selective under acidic conditions and cleaves the second amide bond upstream from the Met residue. However, the same complex is sequence-specific under neutral conditions and cleaves the amide bond only at the Pro-Met or Sar-Met residue with no cleavage observed at Gly-Met peptide (Figure 24).

**Figure 25:**
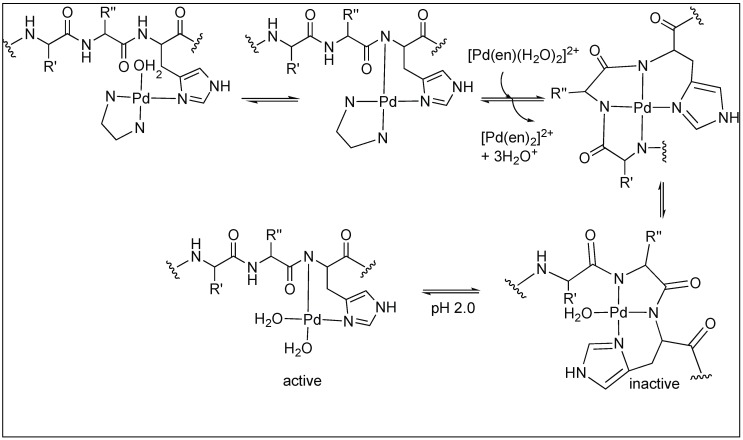
Acidic hydrolysis of amide bonds.

Next, the same group utilized the Pd(en)(H_2_O)_2_ complex for the acidic hydrolysis of the B-chain of bovine insulin containing two histidine residues. These complexes promoted the cleavage of the second amide bond upstream from histidine. The detailed mechanistic analysis showed the selective cleavage by the coordination of the Pd complexes with the histidine side chain and amidic nitrogen followed by the internal attack of water on the amide bond (Figure 25) [101].

**Figure 26:**
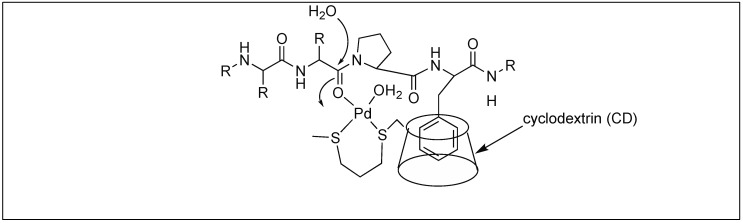
β-Cyclodextrin Pd-complex.

Next, they used the β-cyclodextrin Pd complex for the cleavage of the amide bond at the first amide bond upstream from the Pro-Phe sequence. The role of the β-CD complex was to bind to the hydrophobic residue (Phe) in an aqueous medium followed by the activation of the amide carbonyl group by the metal coordination, and the attack by the external water molecule leading to the cleavage of the amide bond (Figure 26) [102].

#### 3.1.4. Artificial Metal Proteases

**Figure 27:**
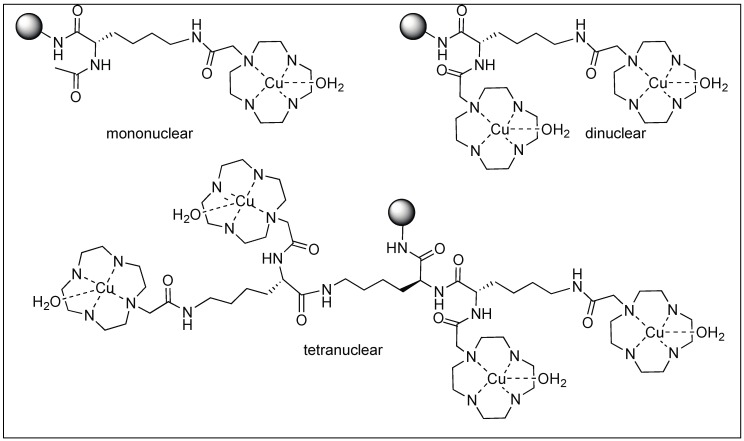
Artificial metal complexes with different numbers of metal centers.

Suh et al. designed metal complexes for the cleavage of amide bonds in proteins at specific locations [103,104,105,106,107]. These metal complexes are highly selective with their protein partners similar to some natural proteases. They also demonstrated that the catalytic rate of hydrolysis increased by the formation of the complex between the substrate and catalyst. This is achieved by increasing the multinuclear metal centers, which provide extra metal centers as substrate binding sites. They showed that the rate of hydrolysis of the myoglobin protein increased with the increase in the number of metal centers in the mono (half-life for hydrolysis 24 h, Figure 27), dinuclear (half-life for hydrolysis 3.5 h), and tetranuclear metal centers (half-life for hydrolysis 1.3 h) [103]. 

They showed that the selectivity towards a specific protein was achieved by the addition of organic pendants such as peptide nucleic acid (PNA), which are responsible for selective binding to a particular protein. Overall, the goal of this research was to synthesize peptide-cleavage agents selective for the hydrolysis of pathogenic proteins responsible for Alzheimer’s disease, type 2 diabetes mellitus, and Parkinson’s disease.

##### Aldehyde Pendant for the Cleavage of Proteins

**Figure 28:**
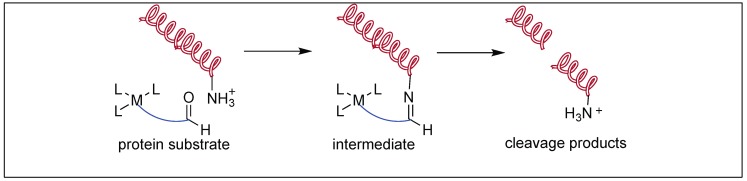
Aldehyde pendant mediated cleavage of peptide bonds.

Ammonium groups are abundant on the surface of proteins and form imine with aldehyde faster than the peptide bond cleavage and, through this process, brings the reactive metal catalytic center in close proximity to the protein/peptide chain (Figure 28). The rapid formation of imine makes the attack of the metal center on the peptide bond an intramolecular process, thus, increases the rate of hydrolysis. Based on this concept, several artificial metalloproteases have been developed by incorporating an aldehyde handle close to the metal center and has been successfully been applied for the faster cleavage of peptide bonds [104].

##### Mb-Selective Artificial Protease

**Figure 29:**
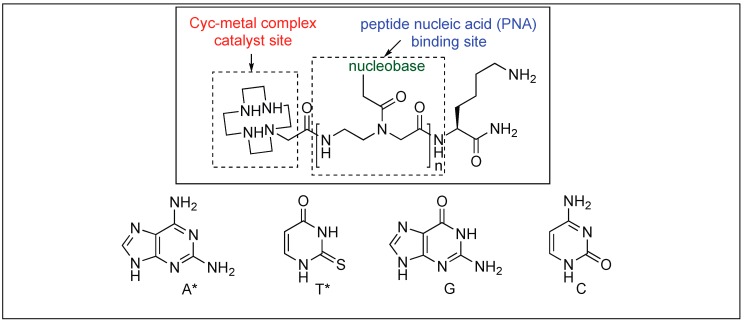
Catalyst design for protein cleavage.

The catalyst for the cleavage of myoglobin (Mb) was designed by attaching the cyclen metal complex containing Cu(II) or Co(III) to the peptide nucleic acid (PNA) monomers, which act as binding sites for the Mb (Figure 29) [105]. Varieties of linkers were inserted between the PNA binding site and the catalytic cyclen site for the formation of Mb-cleaving catalysts. MALDI-TOF MS showed that the cleavage of the peptide backbone chain of Mb takes place at Leu89-Ala90. Various chelating ligands were tested for determining the activity of the Mb-cleaving catalyst but only cyclen and its triaza-monooxo analog showed efficient catalytic activity.

**Figure 30:**
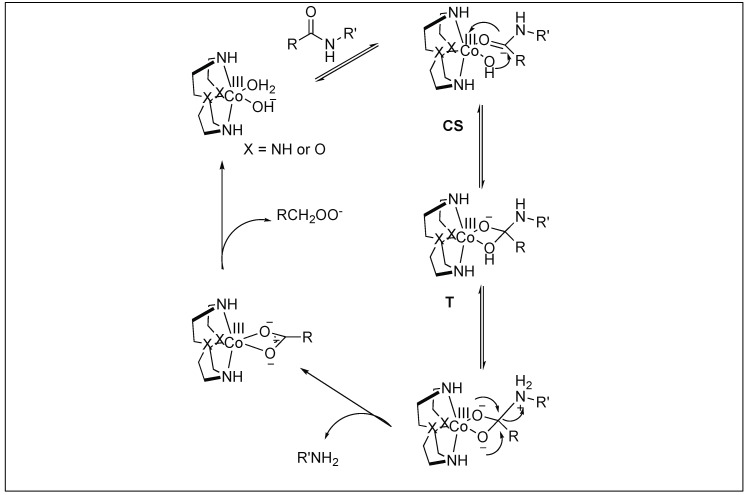
Mechanism of Co(III)-metal complexes.

Mechanism: The first step involves the binding of the carbonyl group of the amide to the Co(III) metal center to form a CS complex (Figure 30). The selectivity of one amide carbonyl over the rest of the amidic carbonyls is based on the other half of the catalyst, which is responsible for the recognition of a particular protein and a particular site. This Co(III) carbonyl coordination activates the amidic carbonyl for a nucleophilic attack by the hydroxide ion on the metal center, resulting in the formation of tetrahedral intermediate T. This is followed by the collapse of the tetrahedral intermediate (T), resulting in the breakage of the amide bond to generate a peptide amine and corresponding peptide acid [106].

Suh and co-workers showed that the cleaving capability of the Co(III)/Cu(II) complexes of cyclen increased by the replacement of one nitrogen atom of cyclen with an oxygen atom. The Co(III)-oxacyclen complexes (1-oxa-4,7,10-triazacyclododecane Co(III)) cleaved the proteins such as BSA, HEWL, Mb, and bovine serum-globulin with a 4–14 times higher catalytic efficiency compared to the Co(III)-cyclen complexes [106].

##### PDF-Selective Artificial Protease

**Figure 31:**
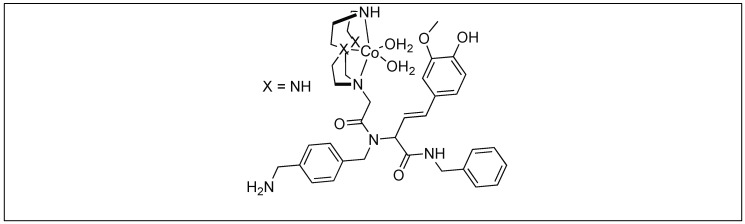
Metal-assisted catalyst for PDF.

The catalyst for the cleavage of the protein, peptide deformylase (PDF), was obtained in a selective manner by screening the library of catalysts (Figure 31). The catalyst cleaved the backbone peptide chain of the PDF at position Gln152-Arg153. Docking simulations showed that multiple interactions were responsible for the formation of a complex between the catalyst and PDF. Fifteen other proteins were examined, but none of them underwent cleavage by this Co(III) complex, which further confirmed the highly selective nature of these metal complexes [107].

##### AmPs-Selective Artificial Protease

**Figure 32:**
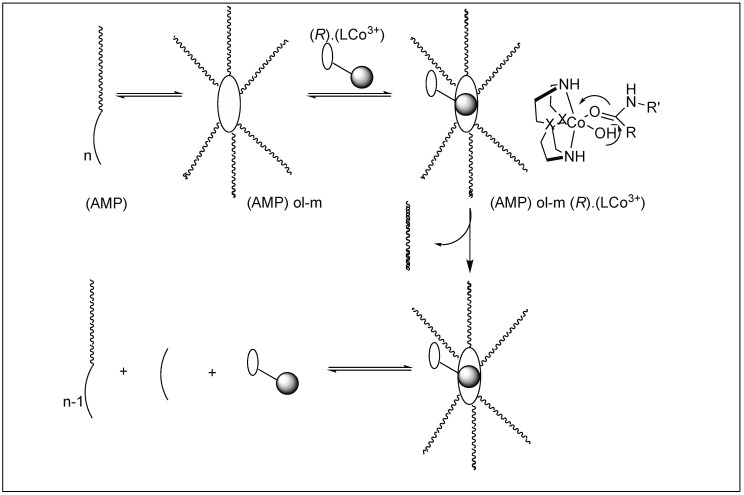
Metal complexes for the cleavage of amyloidgenic peptides.

AmPs are amyloidogenic peptides or proteins which lack active sites and are related to diseases such as Alzheimer’s and type 2 diabetes. Based on the above concept, Suh et al. synthesized various other metal complexes for the selective cleavage of amide bonds in these proteins. The main advantage of this work is that such amyloidogenic proteins cannot be targeted by conventional approaches due to the lack of an active site (Figure 32) [108,109,110].

**Figure 33:**
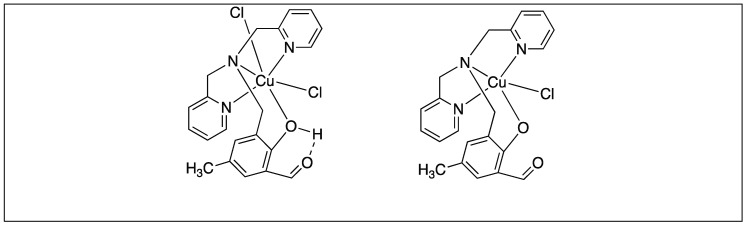
Cu complexes for activation of amide bonds.

Soares et al. utilized mononuclear copper(II) complexes [Cu(HL^1^)Cl_2_] and [Cu(L^1^)Cl] for the cleavage of unactivated amide bonds of the proteins bovine serum albumin (BSA) and Taq DNA polymerase, under mild pH and temperature conditions (Figure 33). The cleavage occurred at the specific site on the solvent- exposed portions of the protein to generate particular proteolytic fragments [111].

### 3.2. The Non-Lewis Acid Reaction Mechanisms Based on the N→O Rearrangement

**Figure 34:**
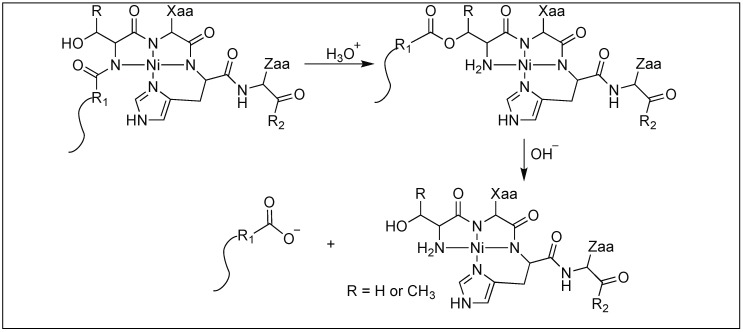
Non lewis acid mediated N, O Acyl rearrangement.

In some cases, metal catalysts showed a high rate of hydrolysis of the peptide backbone chain at the N-terminus of the serine and threonine residues. Such a cleavage was catalyzed by the N→O rearrangement and does not employ the Lewis acid properties of the metal atom. Based on the proposed mechanism, the first step involves the formation of the Ni(II) complex with 4 N of the backbone amide chain and the side chain of the His residue with the (Ser/Thr)-Xaa-His sequence (Figure 34). The second step involves the N→O rearrangement from the side chain of the Ser or Thr that transfers the N-terminal R1 moiety from the peptide bond to form an ester bond. This is followed by the hydrolysis of the resulting ester, leading to the formation of two reaction products, the R1 peptide acid and the Ni(II) complex with the peptide [112,113].

#### Scandium(III) Triflate-Promoted Serine/Threonine Selective Peptide Bond Cleavage

**Figure 35:**
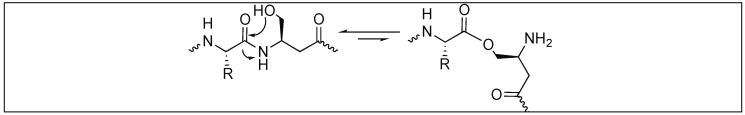
N, O Acyl rearrangement.

Kanai et al. reported the hydrolysis of the peptide bond at the N-terminus of Ser/Thr residue by using scandium triflate. This chemical cleavage relies on Sthe c triflate mediated N to O acyl rearrangement followed by the subsequent hydrolysis of the ester by heating it at 80–100 °C (Figure 35). Complete hydrolysis took place in 18–20 h. The authors have used this approach for the cleavage of various peptides including posttranslationally modified (PTM) peptides and the cleavage of native protein Aβ1-42, which is closely related to Alzheimer’s disease [114].

## 4. Organic Molecules for Activation of Amide Bonds

We have summarized different kinds of nonmetal-based methods, their mechanisms of hydrolysis of unactivated peptide bonds and the point of cleavages in Table 3.

### 4.1. N-Terminal Cleavage of Amide Bonds

#### 4.1.1. Edman’s Degradation 

**Figure 36:**
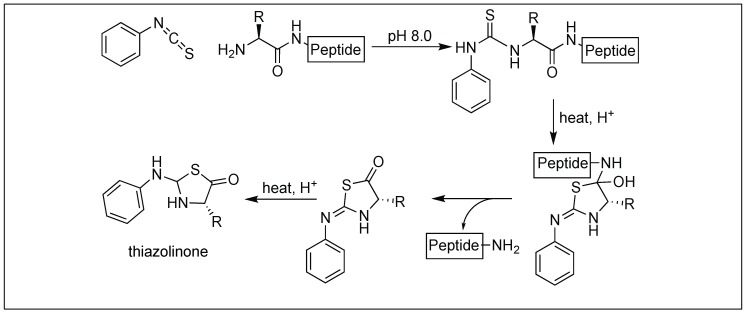
Edman’s degradation approach for cleavage of peptide bonds.

This approach utilizes phenyl isothiocyanate for the cleavage of the peptide bond at the N-terminus. Phenyl isothiocyanate reacted with an uncharged N-terminal amino group, under mildly alkaline conditions, to form a cyclic *phenylisothiocyanate* derivative, which undergoes cleavage as a thiazolinone derivative under acidic conditions (Figure 36). We proposed that the activation of an amide bond is due to the formation of the five-membered cyclic *phenylisothiocyanate* intermediate which creates a twist in the amide bond, thus preventing the amidic nitrogen from forming a resonating structure and making it susceptible towards hydrolysis [115].

#### 4.1.2. Cyanogen Bromide for Cleavage at Met Residue

**Figure 37:**
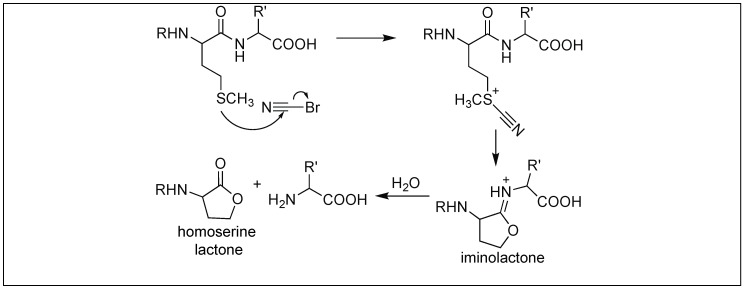
Cyanogen bromide for selective cleavage at Met.

Cyanogen bromide led to the cleavage of the peptide bond at the C-terminus of the methionine residue in a selective manner. The first step involves the nucleophilic attack of the sulfur of methionine on cyanogen bromide (Figure 37) [116]. This displaces the bromide from cyanogen bromide, followed by the attack of the amide carbonyl on the cyano group, resulting in the formation of the five-membered ring, iminolactone, comprising a double bond in the ring between nitrogen and carbon. This double bond results in a rigid ring conformation, thus activating the amide bond towards cleavage at the C-terminus of Met, resulting in the generation of homoserine lactone. This approach has widely been utilized for the sequencing of proteins [116].

#### 4.1.3. 2-Nitro-5-Thiocyano Benzoic Acid for Cleavage at Cys

**Figure 38:**
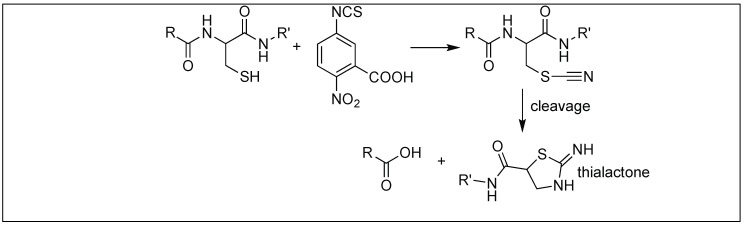
2-nitro-5-thiocyano benzoic acid selective cleavage at Cys.

2-Nitro-5-thiocyano benzoic acid led to the hydrolysis of the amide bond at the N-terminal side of the cysteine residue. The first step is the cyanylation of the side chain of cysteine on a peptide by 2-nitro-5-thiocyano benzoic acid, which is followed by the attack of the cysteine amidic nitrogen to the cyano group on the side-chain of cysteine, resulting in the formation of the 5-membered thiolactone ring. This, in turn, activates the amide bond towards hydrolysis under basic conditions (Figure 38). This is again due to the inability of cysteine amidic nitrogen in a thiolactone to form a resonating structure with the carbonyl of peptide bond [117].

#### 4.1.4. 2-Iodosobenzoic Acid for Cleavage at Trp

**Figure 39:**
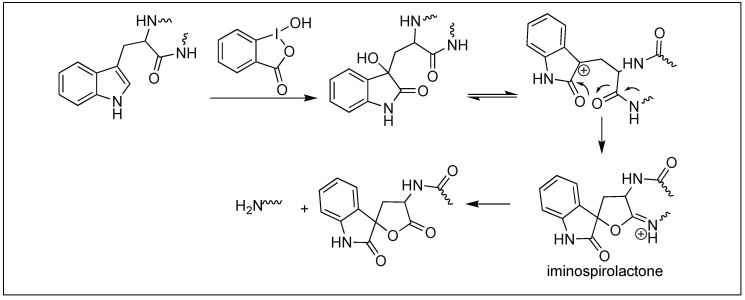
Iodosobenzoic acid for hydrolysis.

2-Iodosobenzoic acid has been used for the hydrolysis of the amide bond at the C-terminal side of the Trp residue. The mechanism of the cleavage is a two-step process. The first step involves the oxidation of the side-chain of tryptophan by 2-iodosobenzoic acid followed by the nucleophilic attack from the neighboring carbonyl group of the amide bond, leading to the formation of an iminospirolactone which hydrolyzes the peptide chain in the presence of water (Figure 39) [118,119].

#### 4.1.5. TBC for Cleavage at Trp 

**Figure 40:**
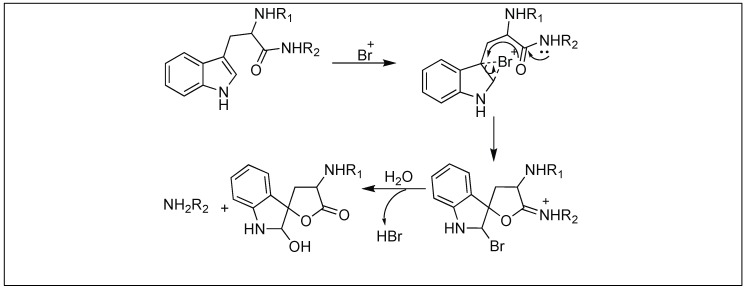
TBC for selective cleavage at Trp residue.

Tryptophanyl peptide bonds underwent selective cleavage by 2,4,6-tribromo-4-methylcyclohexadienone (TBC) at the C-terminus (Figure 40). Tyrosyl and histidyl peptide bonds which are usually cleaved by other brominating agents (such as α-bromosuccinimide, α-bromoacetamide, etc.) are stable to this reagent. Additionally, other amino acids, which are sensitive to oxidation, react with TBC but do not cleave the peptide bonds. This method was successfully applied to a variety of peptides and proteins [118,119].

According to the reaction mechanism suggested by Patchornik et al. (1960), oxidative bromine participates in the modification-cleavage reaction [118,119]. Two equivalents of bromine first brominate the indole nucleus followed by a spontaneous debromination through a series of oxidation and hydrolysis reactions (Figure 40). These reactions led to the formation of an oxindole derivative, which cleaves the peptide bond.

### 4.2. N-Amidination for Cleavage of the N-Terminal Amide Bond

**Figure 41:**
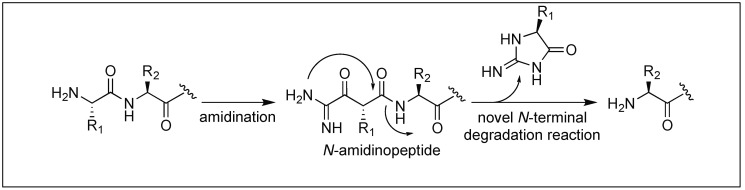
N-amidination strategy.

Hamada et al. reported the cleavage of the amide bonds by the *N*-amidination of peptides. The *N*-amidination of peptides leads to the formation of a cyclic moiety which resulted in the cleavage of the amide bond at room temperature (Figure 41) [120]. The rate of cleavage was slow under ambient conditions (PBS buffer, pH 7.4) at 37 °C with t_1/2_ = 35.7 h, but a rapid cleavage was observed under basic conditions (2% NaOH aq) with t_1/2_ = 1.5 min. To evaluate the broad applicability of this cleavage reaction, a series of peptides with different amino acids at the N-terminus such as the Lys, Glu, Ser, Cys, Tyr, Val, and Pro residues were cleaved with t_1/2_ values from 1 min to 10 min. A slightly slow cleavage was observed with bulky amino acids at the terminus such as Val or Pro, which might be hindering the path of cyclization.

**Figure 42:**
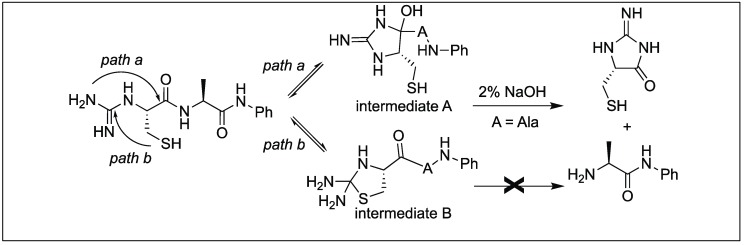
N-amidination strategy with N-terminal cysteine.

*N*-amidinated peptide with a Cys residue at the N-terminus also generated a five-membered ring, thiazolidine by path b (intermediate B) which did not lead to any cleavage, therefore, the t_1/2_ of the peptide with Cys at the N-terminus in 2% NaOH aq at 37 °C was 3.4 min (slower than with other amino acids at the N-terminus) (Figure 42) [120].

### 4.3. Lactonization Mediated Cleavage of Amide Bonds 

Otaka et al. developed an auxiliary with special protecting groups (PGs), which is capable of forming a lactone with the carbonyl of an amide bond, resulting in the cleavage of the amide bond (Table 4) [121,122,123,124,125,126,127,128]. Depending on the nature of the protecting groups, an amide bond cleavage can be initiated in peptides by using different responsive reagents for the deprotection of PGs (Table 4). Table 4 showed various PGs and the corresponding responsive reagents for their deprotection. The thiol responsive reagent was applied for the cleavage of the PNA/DNA complex using thiol-responsive protecting groups [121,122,123,124,125,126,127,128]. 

### 4.4. Hydrogen Peroxide-Induced Amide Bond Cleavage

**Figure 43:**
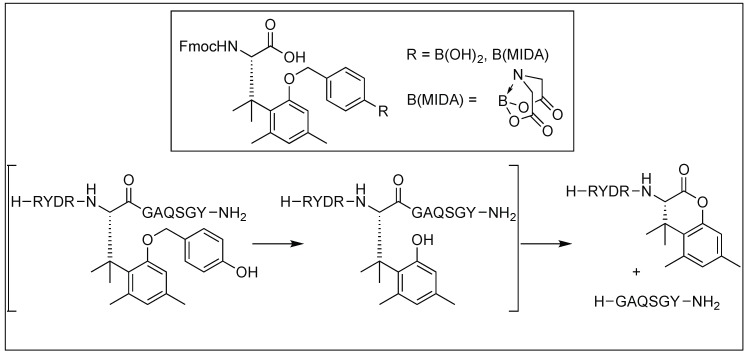
Hydrogen peroxide responsive probes.

Later, the hydrogen peroxide (H_2_O_2_)-responsive protecting group was introduced to the amino acid. This protecting group contains a boronate or boronic acid moiety which underwent deprotection in oxidative stress because of the release of hydrogen peroxide followed by the formation of lactone and the cleavage of the peptide bond (Figure 43) [128].

### 4.5. Glutamic Acid Specific Activation of Amide Bonds

**Figure 44:**
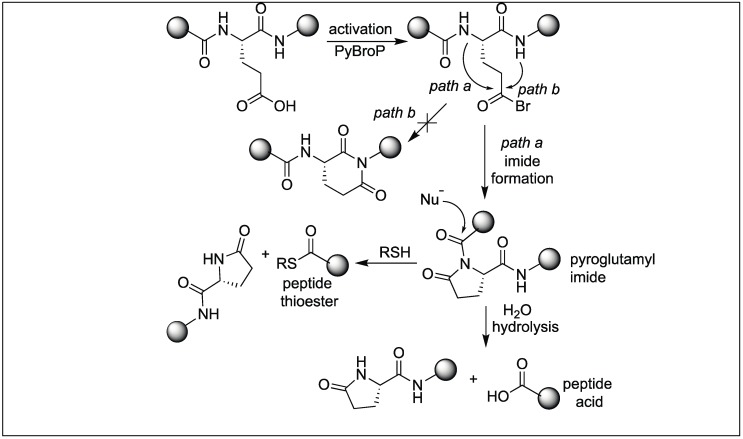
Glutamic acid selective activation of peptide bonds.

We have also reported a new method for the site-specific cleavage of peptide bonds at glutamic acid under physiological conditions [129,130]. The method involves the activation of the backbone peptide chain at the N-terminal side of glutamic acid by the formation of a pyroglutamyl imide (pGlu) moiety using bromotripyrrolidinophosphonium hexafluorophosphate (PyBroP) (Figure 44). This activation increases the susceptibility of the peptide bond toward various nucleophiles including thiol and water (Figure 44). We showed that this pyroglutamyl imide activated peptide chain underwent the complete cleavage of the peptide bond under neutral buffer conditions (pH 7.5). It was observed that the rate of hydrolysis increase under basic pH conditions (pH = 10.5). Although the Asp has a carboxylic group on the side chain, no cleavage was observed under the reaction conditions. Jensen et al. exposed the pGlu activated peptide bond towards thiol, resulting in the formation of peptide thioesters [129,130]. A noted feature about this approach is that it leads to the formation of epimerization free peptide acids and peptide thioesters. This method is highly specific and exhibits a broad substrate scope including the cleavage of bioactive peptides with unnatural amino acids, which are unsuitable substrates for enzymes.

### 4.6. Asparagine Selective Cleavage of Amide Bonds

**Figure 45:**
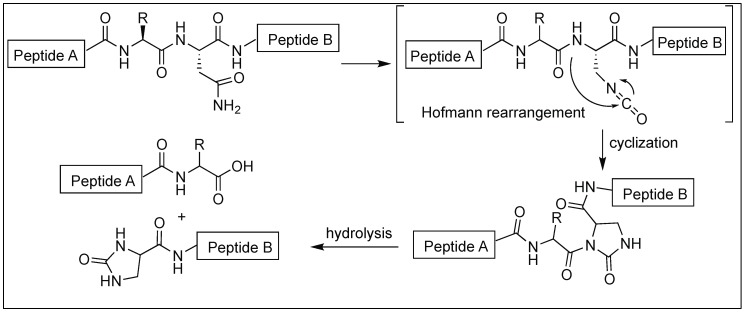
Asparagine selective cleavage of peptide bonds.

Kanai et al. described the method for the site-selective chemical activation of peptide bonds for hydrolysis at the asparagine residue using diacetoxyiodobenzene (DIB) [131]. The reaction of the side-chain of Asn with DIB leads to the formation of isocyanate by Hofmann rearrangement. This is followed by the attack of the N-terminal amidic nitrogen of the peptide backbone chain, affording a five-membered *N*-acylurea intermediate, thus activating the amide bond towards hydrolysis (Figure 45). Asn-selective peptide bond cleavage was proceeded in an aqueous neutral solution at 37 °C and exhibited a broad substrate scope. The Gln-site was not cleaved under the reaction conditions. Specifically, this method is applicable to peptides containing unnatural amino acids and/or posttranslational modifications where enzymatic cleavage is not very efficient.

### 4.7. Cyclic Urethane Mediated Activation of Amide Bonds

**Figure 46:**
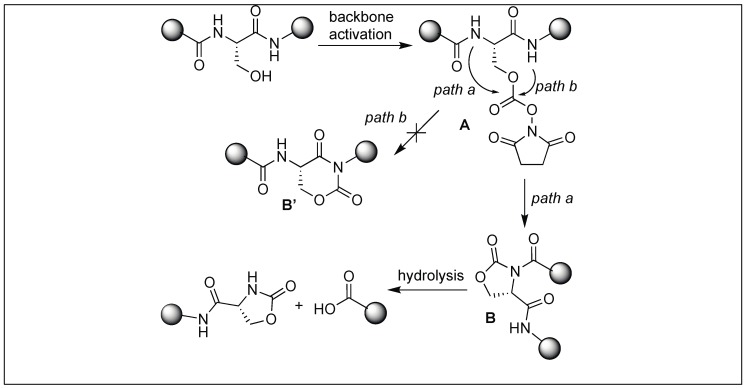
Cyclic urethane mediated activation of peptide bond.

We have developed a method for the cleavage of the amide backbone chain at the N-terminal side of Ser, Thr, and Cys by the formation of a five-membered cyclic urethane moiety [132]. The formation of the cyclic urethane moiety with an amide backbone makes the amidic carbonyl group susceptible to nucleophilic attack. This is presumably due to the twist in the backbone amide chain caused by the cyclic urethane moiety. Thus, it was no longer able to form a resonating structure. To achieve this goal, we screened various carbonylating reagents and a maximum conversion to cyclic urethane moiety was achieved with *N*,*N*-disuccinimidyl carbonate (DSC). We proposed that the hydroxymethyl group of the side chain of Ser reacted with DSC to generate an activated intermediate, A, which then undergoes nucleophilic displacement by the amidic nitrogen on the N-side of serine through the path to generate a five-membered cyclic urethane intermediate B (Figure 46). The formation of the cyclic urethane intermediate B makes the amide bond susceptible to nucleophilic attack and led to the cleavage of the amide bond in neutral aqueous conditions (room temp, pH 7.5, 12 h). There is a possibility of nucleophilic displacement of the intermediate A by the amidic nitrogen through path b. This could lead to the formation of six-membered ring B′, but we did not observe the formation of any six-membered ring as analyzed by NMR studies. 

**Figure 47:**
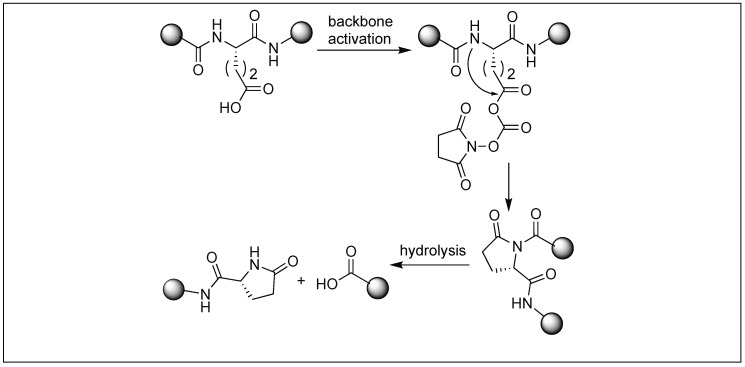
Pyroglutamyl imide mediated activation of peptide bond.

**Figure 48:**
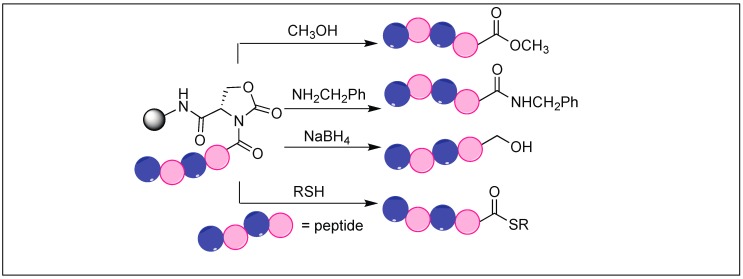
Cyclic urethane mediated synthesis of C-terminal peptides.

The side chain of Glu on reaction with DSC also led to the formation of a pyroglutamyl imide moiety with an amide backbone chain, thus making it susceptible to nucleophilic attack (Figure 47) [132]. We have used this approach for the selective hydrolysis of peptides/proteins at the N-terminus of Ser, Thr, Cys, and Glu. This method cleaved various bioactive peptides containing posttranslational modifications (e.g., *N*-acetylation and -methylation) and mutations (d-and β-amino acids), which are not suitable substrates for enzymes, thus exhibited a broad substrate scope. We have also used this approach for the synthesis of a variety of functionalized C-terminal peptides such as esters, amides, alcohols, and thioesters (Figure 48) by exposing the cyclic urethane activated peptide towards various nucleophiles such as alcohols, amines, reducing agents, and thiols [133,134]. The attractive feature of this approach is that it leads to the formation of epimerization free C-functionalized peptides. 

**Figure 49:**
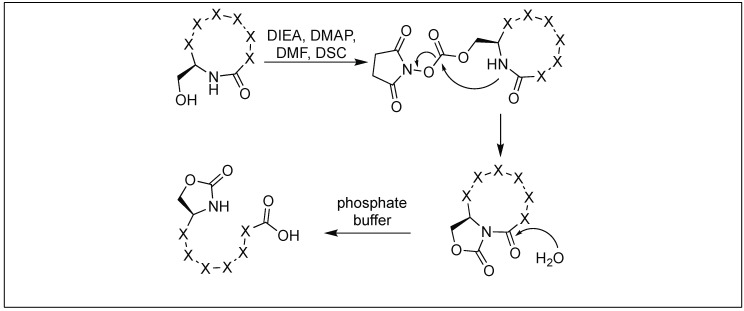
Cyclic urethane for cleavage of cyclic peptides.

**Figure 50:**
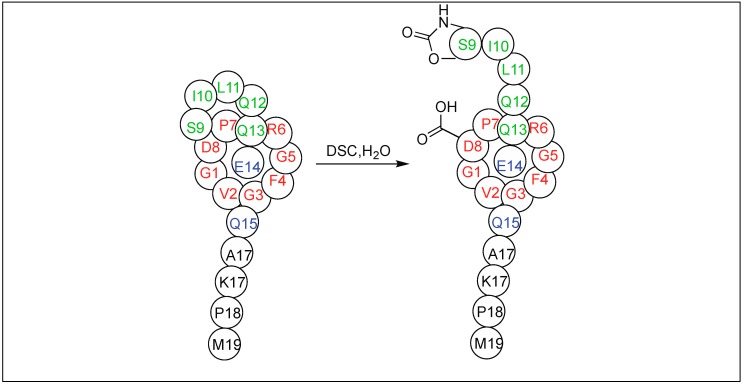
Synthesis of rotaxane from lasso peptide.

Later, this cyclic urethane amide-activation approach was applied for the cleavage of a variety of cyclic and lasso peptides obtained from nature to determine their sequence, which is difficult to be determined by conventional approaches (Figure 49) [135,136]. We have also applied this method for the synthesis of a peptide-based molecular machine (rotaxanes) for the first time (Figure 50) [135,136].

### 4.8. Intein-Inspired Amide Cleavage Chemical Device

A photoresponsive device was developed for the cleavage of the amide bond at the C-terminus of the Asn residue [137]. This approach was inspired by intein-mediated protein splicing and its chemical environment was mimicked by the incorporation of geminal dimethyl groups and a secondary amine on the asparagine scaffold. 

**Figure 51:**
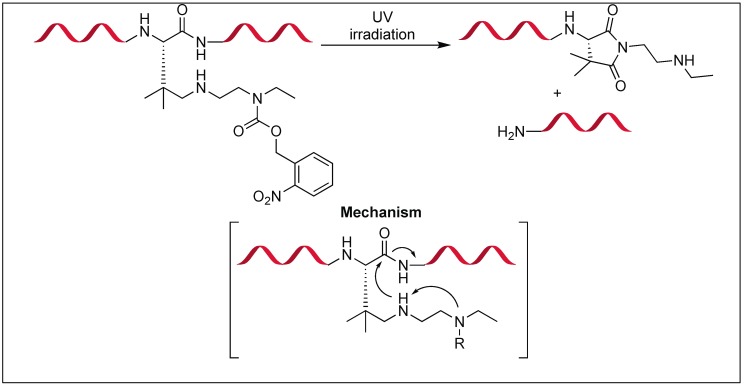
Intein-inspired amide bond cleavage.

The secondary amine acts as an intramolecular base, which enhances the nucleophilicity of the amide nitrogen (Figure 51). The geminal dimethyl groups led to a Thorpe-Ingold effect, which enhances the intramolecular attack, thus assisting in the formation of the succinimide ring [138,139,140]. The *o*-nitrobenzyloxycarbonyl (*o*-NBnoc) masks the basic character of the secondary amine [141,142], thus leading to the photo triggered cleavage of an amide bond by the deprotection of the secondary amine unit containing the *o*-nitrobenzyloxycarbonyl group. 

### 4.9. Serine-Selective Aerobic Cleavage of Peptides 

Kanai et al. reported a use of the water-soluble copper-organoradical conjugate for the selective cleavage of the peptide bond at the N-terminus of a serine residue under mild conditions and at room temperature [143]. They used this approach for the selective cleavage of a variety of different peptides/proteins containing D-amino acids or sensitive disulfide pairs. 

**Figure 52:**
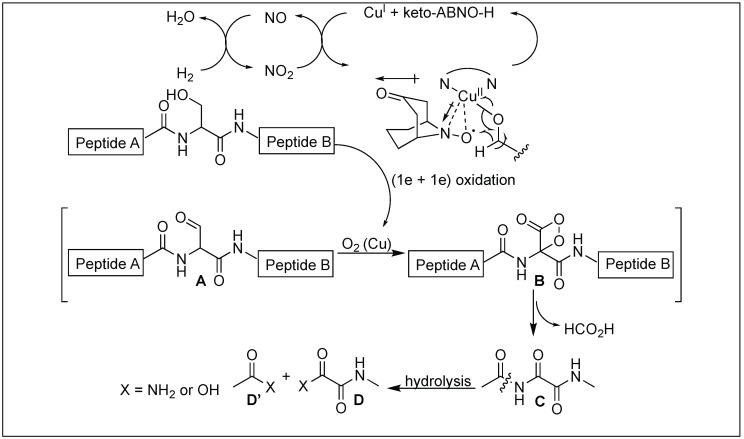
Serine selective aerobic cleavage of peptide bonds.

Ser-selective cleavage of the peptide bond was initiated by the aerobic chemoselective oxidation of the hydroxymethyl moiety of Ser to a formyl group (**A**) (Figure 52). This produced a β-formyl glycineamide intermediate **B** which, on further oxidation, led to the formation of oxalamide **C** by undergoing oxidative deformylation. Oxalamide **C** then underwent hydrolysis under mild conditions because the carbonyl groups of the oxalamide are more electrophilic than those of simple amides, resulting in the formation of the cleaved fragments **D** and **D′**. By using molecular oxygen as a terminal oxidant, water and a C1 molecule (possibly HCO_2_H) become stoichiometric side products. This strategy is widely distinct from Lewis acid, promoted by the Ser-selective peptide hydrolysis through N-to-O rearrangement.

### 4.10. Hydrolysis of Amide Bonds by the Formation of Oxazolinium Specie: Function of Acyl Protecting Group

**Figure 53:**
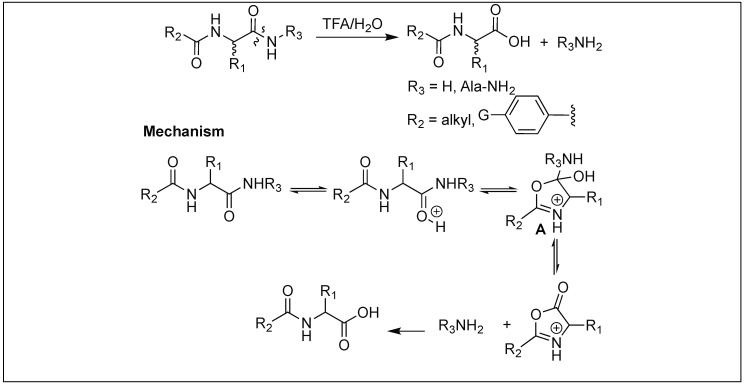
Oxazolinium species formation.

Peptides containing a simple *N*-acyl group activates the amide bond four bonds away from an acyl group for cleavage under acidic conditions [144]. First, TFA leads to the protonation of amide carbonyl followed by the nucleophilic attack from the oxygen of the acyl carbonyl to generate a five-membered oxazolinium specie **A** in the peptide chain. Second, the collapse of the oxazolinium intermediate **A** leads to the cleavage of an unactivated amide bond (Figure 53). 

The nature of the aromatic group, G, was responsible for the rate of hydrolysis of the peptide bond. Electron-donating groups increase the rate of hydrolysis whereas electron withdrawing substituents decrease it (Figure 53).

### 4.11. Hydrazinolysis for the Cleavage of Amide Bonds

**Figure 54:**
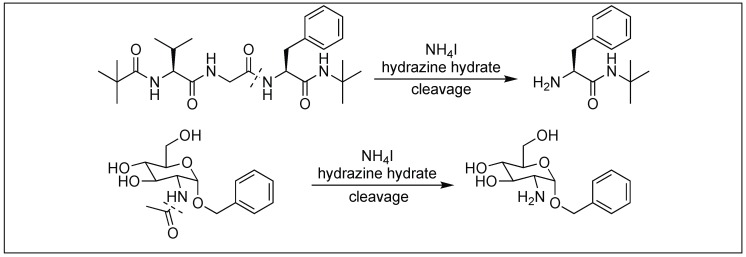
Hydrazinolysis for the cleavage of peptide bonds.

The hydrazinolysis of unactivated amide bonds was accelerated by the addition of ammonium salts. The reaction proceeds at 50–70 °C to give peptide cleavage products and exhibits a broad substrate scope that out-performs existing amide bond cleavage reactions [145]. This approach was applied for the cleavage of the peptide bonds without racemization at the α-position of the amino acids (Figure 54). It was also applied to the cleavage of the *N*-acetyl group from the amino sugar.

### 4.12. Amide Bond Cleavage of the N-Methylcysteinyl Peptide

**Figure 55:**
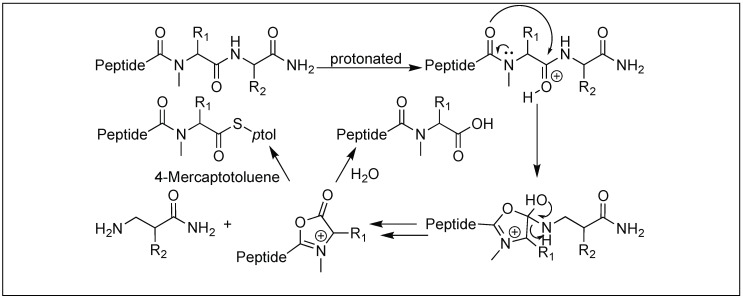
*N*-methylcysteinyl peptide cleavage.

Tam et al. developed a selective bi-directional peptide bond cleavage approach utilizing *N*-methylcysteine (MeCys) in the Xaa-MeCys-Yaa peptides (Xaa and Yaa, non-cysteine residues) [146]. Under strong acidic conditions, peptide Xaa-MeCys-Yaa led to the formation of an oxazolinium intermediate, resulting in the cleavage of the Xaa-MeCys bond. The oxazolinium intermediate was later trapped by thiocresol (TC) to form a Xaa-MeCys-TC thioester (Figure 55). The replacement of MeCys by Cys residue did not result in the peptide bond cleavage, suggesting the important role of *N*-methylation in MeCys residue for the formation of oxazolone.

### 4.13. N-MeAib Induced Unusual Cleavage of Amide Bonds

**Figure 56:**
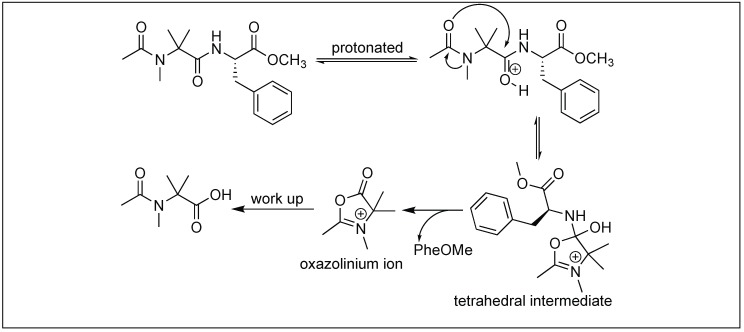
N-Me Aib mediated amide bond cleavage.

Peptides containing acylated *N*-methyl-aminoisobutyryl (NMeAib) residues showed unusual cleavage of amide bonds under acidic conditions. The cleavage takes place at the C-terminal side of the NMeAib residue (Figure 56) [147]. X-ray diffraction studies of the NMeAib containing molecules showed that the oxygen atom of the carbonyl group of the preceding residue is close to the carbonyl carbon of the NMeAib residue, thus acting as an internal nucleophile forming a tetrahedral intermediate. Once this tetrahedral intermediate was formed, lone pair electrons on the nitrogen of the phenylalanine were no longer be able to form resonating structures with the carbonyl group of NMeAib. In fact, the phenylalanine nitrogen becomes a proton acceptor like the amines, which resulted in the cleavage of the amide bond followed by the removal of phenylalanine and the formation of an oxazolinium ion intermediate, which further reacts with water to form a carboxylic acid product.

## 5. Conclusions

This mini-review highlights the methods for the activation of unactivated amide bonds in biomolecules. This review further highlights the application of these methods in the sequencing of proteins and the synthesis of peptide acids, thioesters, alcohols, and amides. These studies showed that there is still a lot to learn from enzymes catalyzed pathways and how we can develop enzyme mimetics for catalyzing the cleavage of unactivated and highly stable amide bonds at particular residues in a selective manner. We assume that these enzyme mimetics can have potential applications in various fields. 

## Figures and Tables

**Table 1:** Enzymatic directed hydrolysis of peptide bonds.

Entry	Enzyme	Method of Hydrolysis	Point of Cleavage	Ref.
1	Serine and Cysteine Proteases	Oxyanion binding hole with catalytic triad	-	[32,33,34,35,36,37]
2	Metallo-endopeptidase	Thermolysine with Zn^2+^ binds to His 142, His 146, Glu 166	Internal peptide bonds on the N-terminal side of large hydrophobic amino acids	[38,39,40,41,42,43,44,45,46,47,48,49,50,51]
3	Metalloexopeptidase	Carboxypeptidase A with Zn^2+^ through Lewis acid activation	C-terminus comprising large hydrophobic amino acids	[52,53,54]
4	*O*-GlcNAc transferase	Glycosylation followed by enzyme catalyzed pyroglutamate formation	N-terminal glutamic acid	[55,56]
5	Nicotinamidase	Enzyme Chelation to Zn^2+^ and catalytic triad	Nicotinamide	[57,58,59,60]
6	Flavoenzyme	Flavin hydroperoxide intiated oxidative mechanism	Unactivated amide bond in uracil	[61,62,63]
7	Antibody Fab-BL 125	Catalyzes unactivated primary amide bond hydrolysis	Primary amide bond of l-isomer of peptides	[64,65]
8	RNA	Mg^2+^ catalyzed mechanism	Unactivated alkyl amide of DNA analog	[66]

**Table 2:** Metal catalyzed hydrolysis of peptide bonds.

Entry	Metal Complex	Method of Hydrolysis	Point of Cleavage	Ref.
1	Simple metal ions	Lewis acidity of metal ion	C-terminal of peptide	[67,68,69,70,71,72]
2	Zr POMs, Zr MOF-808	Lewis acidity of metal ion	C-terminal of peptide	[73,74,75,76,77,78,79,80,81,82]
3	Mo(VI)	Lewis acidity of metal ion and formation of 5 membered ring	C-terminal side of Asp	[81,82]
4	Co(III)	N-terminal amine intiated tertiary complex	C-terminal of peptide	[83,84,85,86,87,88]
5	Mo(II)	Favorable six-membered chelate ring	C-terminal side of Cys	[89]
6	Pd(II), Pt(II)	Carboxylic group of amino acid and side chain of amino acid anchoring metal complex	C-terminal side of Met, His, Cys and S-MeCys	[90,91,92,93,94,95,96,97,98]
7	Pd(0)	Methionine side chain anchoring metal complex	Second amide bond upstream from Met	[99,100,101,102]
8	Co(III) and Cu(II)	Lewis acidity activation and PNA for selectivity towards a particular protein	Mb = Leu 89 − Ala 90	[103,104,105,106,107,108,109,110,111]
			PDF = Gln 152 − Arg 153	
			BSA = Solvent exposed portion	
9	Ni(II)	Non Lewis acid based N,O acyl rearrangement	N-terminal side of Ser/Thr	[112,113]
10	Sc(III)	Lewis acid based N,O acyl rearrangement	N-terminal side of Ser/Thr	[114]

**Table 3:** Organic molecules based hydrolysis of peptide bonds.

Entry	Organic Molecules	Method of Hydrolysis	Point of Cleavage	Ref.
1	Phenyl isothiocyanate	Through 5 membered cyclic phenylisothiocyanate intermediate	N-terminal side of peptide	[115]
2	Cyanogen bromide	Through 5 membered iminolactone	C-terminal side of Met	[116]
3	2-Nitro-5-thiocyano benzoic acid	Through 5 membered thialactone	N-terminal side of Cys	[117]
4	2-iodosobenzoic acid	Through iminospirolactone	C-terminal side of Trp	[118,119]
5	TBC	Through Oxindole	C-terminal side of Trp	[118,119]
6	*N*-amidination	Through 5 membered cyclic amidine ring	N-terminal side of peptide	[120]
7	Protecting goups (Table 4)	Lactonization strategy	C-terminal side of peptide	[121,122,123,124,125,126,127]
8	H_2_O_2_ responsive protecting groups	Lactonization strategy	C-terminal side of peptide	[128]
9	PyBroP	Glutamic acid selective activation through pyroglutamyl imide	N-terminal side of Glu	[129,130]
10	DIB	Hofmann rearrangement mediated *N*-acylurea intermediate	N-terminal side of Asn	[131]
11	DSC	Cyclic urethane amide activation	N-terminal side of Ser, Thr, Cys and Glu	[132,133,134,135,136]
12	*o*-NBnoc	A photo responsive amide cleavage through succinimide ring	C-terminal side of Asn	[137,138,139,140,141,142]
13	Cu-organo radical conjugate	Aerobic chemoselective oxidation of Ser followed by oxalimide formation	N-terminal side of Ser	[143]
14	TFA/H_2_O	N-acyl group mediated oxazolinium specie	C-terminal side of peptide	[144]
15	Hydrazine hydrate	Hydrazinolysis accelarated by addition of ammonium salts	N-terminal of peptide	[145]
16	*N*-Mecysteine	Through oxazolinium ion	C-terminal side of *N*-MeCys	[146]
17	Acylated *N*-MeAib	Through oxazolinium ion	C-terminal side of *N*-MeAib	[147]

**Table 4:**
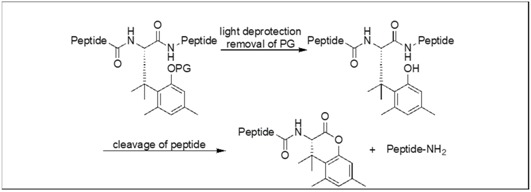
Lactonization mediated cleavage of amide bonds.

Reagent/Condition	PG	R1	R2	R3
Ultraviolet Near-infrared Fluoride hypoxia	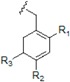	NO_2_	H	H
		NO_2_	OMe	OMe
		H	OTBDPS	H
		H	NO_2_	H
Thiol	4-nitrobenzenesulfonyl	-	-	-
Phosohatase	phosphate	-	-	-
